# Health-related quality of life and its risk factors in Chinese hereditary angioedema patients

**DOI:** 10.1186/s13023-019-1159-5

**Published:** 2019-08-08

**Authors:** Shuang Liu, Xue Wang, Yingyang Xu, Qun Xu, Yuxiang Zhi

**Affiliations:** 1 Department of Allergy, Peking Union Medical College Hospital, Chinese Academy of Medical Sciences and Peking Union Medical College, Beijing, 100730 China; 2 School of Clinical Medicine, Chinese Academy of Medical Sciences and Peking Union Medical College, Beijing, China; 3 Key Laboratory of Endocrinology of National Health Commission, Department of Endocrinology, Peking Union Medical College Hospital, Chinese Academy of Medical Science and Peking Union Medical College, Beijing, 100730 China; 4 Department of Epidemiology and Biostatistics, Institute of Basic Medical Sciences Chinese Academy of Medical Sciences, School of Basic Medicine, Peking Union Medical College, Beijing, 100005 China; 5 Center of Environmental and Health Sciences, Chinese Academy of Medical Sciences, Peking Union Medical College, Beijing, 100005 China

**Keywords:** Hereditary angioedema, Health-related quality of life, Chinese, SF-36

## Abstract

**Background:**

Hereditary angioedema (HAE) is a rare but serious condition characterized by unpredictable and recurrent attacks affecting the skin and mucosa. HAE has wide-ranging impacts on the health-related quality of life (HRQoL) of patients. This study aims to assess the HRQoL of Chinese patients with HAE using the 36-item Short Form Health Survey (SF-36v2) and to explore potential risk factors for low HRQoL.

**Methods:**

A total of 104 patients (47 male and 57 female) over age 18 living in China with a known diagnosis of HAE due to C1-INH deficiency completed the SF-36v2 (generic HRQoL questionnaire). The results were compared to Chinese population norms. Subgroup analysis and logistic regression were used to interpret the data.

**Results:**

SF-36v2 showed a significant reduction in all dimensions of HRQoL (*p* < 0.001) in patients with HAE compared with the general Chinese population. Female patients reported significantly lower bodily pain (BP) (*p* = 0.039) and physical component scores (PCSs) (*p* = 0.027) than male patients. Patients with mucosal edema tended to report lower role-physical (RP) limitations (*p* = 0.031) than patients with only skin edema. There were no differences between the mean scores of the SF-36 in relation to disease subtype, age, disease severity and long-term prophylaxis. Among female patients on long-term prophylaxis, social functioning (SF) (r = − 0.404, *p* = 0.010), role-emotional (RE) (r = − 0.320, *p* = 0.044) and mental component scores (MCSs) (r = − 0.313, *p* = 0.049) were negatively correlated with danazol dosage. A correlation between decreased disease control and decreased HRQoL scores was found, although the correlation was not significant in terms of RE or mental health (MH) scores. The logistic regression model revealed uncontrolled disease to be a risk factor for a low PCS (odds ratio 10.77, 95% confidence interval [CI] 1.78–65.06; *p* = 0.010) and laryngeal edema to be a risk factor for a low MCS (odds ratio 4.75, 95% CI 1.09–20.69; *p* = 0.038).

**Conclusions:**

Chinese HAE patients reported significantly lower HRQoL scores than the general population. Unsatisfactory disease control is a risk factor for decreased PCSs. Laryngeal edema is a risk factor for decreased MCSs.

**Electronic supplementary material:**

The online version of this article (10.1186/s13023-019-1159-5) contains supplementary material, which is available to authorized users.

## Introduction

Hereditary angioedema (HAE), a rare autosomal dominant disorder, is characterized by unpredictable and recurrent attacks of painful swelling that typically affect the extremities, bowel mucosa, genitals, face and upper airways [1]. Several forms of HAE have been defined: [1] type 1 HAE (HAE-1), which is caused by C1 inhibitor (C1-INH) deficiency and characterized by low C1-INH level and function; [2] type 2 HAE (HAE-2), which results from C1-INH dysfunction and is characterized by normal or slightly higher C1-INH levels but impaired function; and [3] HAE with normal C1 inhibitor level and function, which is caused by a mutation in the F12 gene (HAE-FXII), the angiopoietin-1 gene (HAE-ANGPT1), in the plasminogen gene (HAE-PLG) and in unknown genes (HAE-UNK) [2]. The pathogenesis remains unknown in some HAE patients. Inherited in an autosomal dominant manner, HAE due to C1-INH (C1-INH HAE) has an estimated prevalence of 1.1–1.6/100,000 with no gender difference [3]. The variability in disease manifestations and difficulties in differentiating HAE symptoms from those of more common angioedema subtypes account for a tendency of misdiagnosis and lead to a diagnostic delay typically longer than 10 years [4]. In addition, a considerable proportion of patients experience potentially lethal laryngeal edema as the disease progresses [5]. Therefore, HAE poses a serious threat to a patient’s life expectancy and quality of life.

Health-related quality of life (HRQoL) is defined as the subjective perception by patients of the multidimensional impacts of a disease or condition [6]. HRQoL assessments could provide patients, healthcare providers and decision makers with more comprehensive information concerning patient health status, disease burden and therapeutic response. A series of validated questionnaires has been used to measure the HRQoL of HAE patients, including generic assessments (e.g., the Short Form 36 Health Survey [SF-36] [7]) and specific questionnaires for a certain disease or condition (e.g., the Angioedema Quality of Life Questionnaire [AE-QoL] [8]). Generic tools make it possible to compare the HRQoL between different diseases, while specific questionnaires are better at reflecting certain characteristics of a particular disease or condition.

HAE produces wide-ranging impacts in patients across physical health (PH) and mental health (MH) domains. Its unpredictable edematous attacks can cause disfigurement and severe bodily pain (BP), thereby negatively impacting the educational attainment, career advancement and social activity of patients [9]. Laryngeal edema can lead to upper airway obstruction, asphyxiation and even death [10]. Gastrointestinal attacks, which cause severe pain, are often mistaken for acute abdomen, resulting in unnecessary surgical interventions [11]. In addition, increased levels of depression and anxiety have been reported by several studies [12, 13]. Bonner et al. proposed a patient-reported outcome (PRO) in which the impacts of HAE attacks on different aspects of life were elaborated [14].

The first HRQoL study for patients with HAE was published in 1999; in this study, the Dermatology Life Quality Index (DLQI) was explored to measure the disability in different urticarial groups, including C1-INH-HAE patients [15]. Short Form Health Surveys (including SF-12 and SF-36) are the most widely applied tools in generic HRQoL assessments. Lumry et al. used SF-12 to evaluate HRQoL in US C1-INH-HAE patients and found impaired PH and MH in comparison to those of the general population [9]. Studies in France [16], Sweden [17], Denmark [18], Brazil [19], Canada [7], the United Kingdom [20] and Colombia [21] have reached similar conclusions. In addition, increasingly more therapeutic studies have incorporated HRQoL assessments in the outcome measures in recent years [22–24]. Caballero et al. summarized the main research findings concerning HRQoL studies on HAE patients in 2017 [6].

Despite the emergence of numerous treatment advances, until now, no acute attack medication has been approved in China. The management is limited to fresh frozen plasma (FFP) transfusion and long-term prophylaxis with danazol or tranexamic acid. Therefore, studies in other countries do not necessarily represent the current state of the HRQoL in China. The HRQoL status in Chinese HAE patients has not yet been satisfactorily described. The aim of this study was to assess the HRQoL of Chinese patients with HAE using the 36-item Short Form Health Survey (SF-36v2) and to explore potential risk factors for decreased HRQoL.

## Results

### Demographic and clinical characteristics of the patients

One hundred and four patients completed this study. Baseline demographic and clinical characteristics are shown in Table 1.

**Table 1:** Baseline characteristics of the 104 hereditary angioedema patients

	N (%) or median [IQR]
Total	104 (100.0)
C1-INH HAE diagnosis	
Type 1	101 (97.1)
Type 2	3 (2.9)
Sex	
Male	47 (45.2)
Female	57 (54.8)
Age (years)	
18–30	27 (26.0)
31–44	52 (50.0)
≥ 45	25 (24.0)
Age at symptom onset (years)	
0–10	16 (15.4)
11–20	54 (51.9)
≥ 21	34 (32.7)
Annual attack frequency (*n* = 75)	3.0[0.8–6.0]
Attacks in the 4 weeks before HRQoL measurement	
Positive	49 (47.1)
Negative	55 (52.9)
Current skin edema	
Positive	81 (77.9)
Negative	23 (22.1)
Current GI edema	
Positive	58 (55.8)
Negative	46 (44.2)
Current laryngeal edema	
Positive	26 (25.0)
Negative	78 (75.0)
Severity evaluation	
Mild	39 (37.5)
Moderate	20 (19.2)
Severe	45 (43.3)
Disease control status	
Completely controlled	35 (33.6)
Partly controlled	55 (52.9)
Not controlled	14 (13.5)
Long-term prophylaxis	
Positive	74 (71.2)
Negative	30 (28.8)
Family history	
Positive	72 (69.2)
Negative	32 (30.8)

### Scores of SF-36 in Chinese HAE patients

**Fig. 1:**
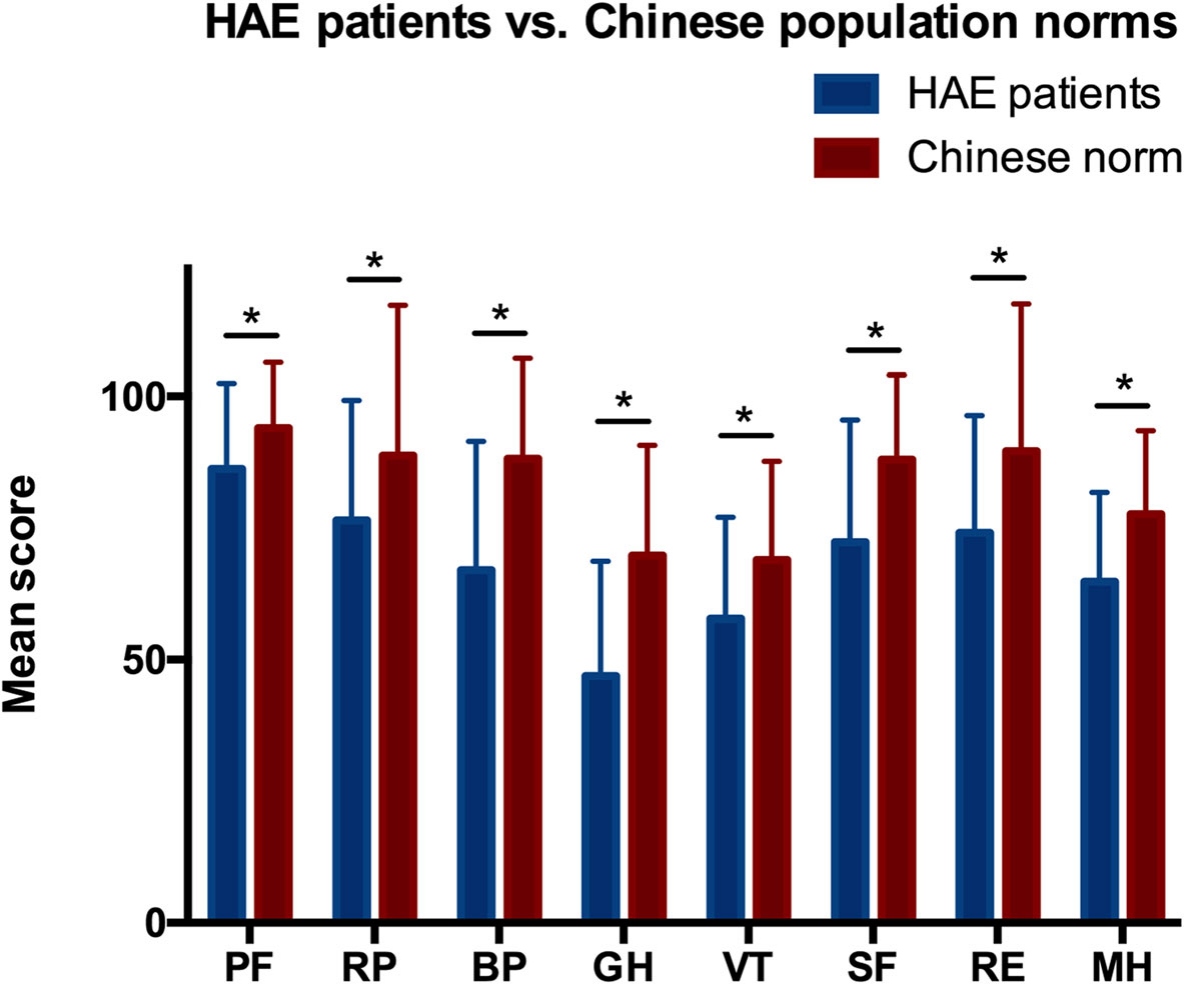
Mean scores in SF-36v2 for Chinese hereditary angioedema (HAE) patients vs. Chinese population norms. *PF* physical functioning, *RP* role-physical, *BP* bodily pain, *GH* general health, *VT* vitality, *SF* social functioning, *RE* role-emotional, *MH* mental health. * *p* < 0.001

Figure 1 outlines the SF-36 mean score for 8 specific dimensions. In all dimensions, HAE patients score significantly lower than the general population (Fig. 1). The physical component score (PCS) and mental component score (MCS) of participants are 49.81 ± 7.08 and 44.76 ± 9.18, respectively. Scores of each dimension are shown in Additional file 1: Table S1.

Female patients reported lower scores than male patients in all eight dimensions and two summary component scores; however, only the differences in terms of BP (*p* = 0.039) and the PCS (*p* = 0.027) were significant.

When we stratified the patients into patients with mucosal edema and those without, a significant difference was observed in terms of RP. Patients with mucosal edema tended to report lower RP (*p* = 0.031) than patients with only skin edema. Compared to patients with only laryngeal or gastrointestinal edema, those with both laryngeal and gastrointestinal edema did not show significant differences in any dimension or summary score.

No significant differences were observed between type 1 and 2 patients. When stratifying patients into three subgroups according to age, i.e., 18–30, 31–44, and over 45, we found no significant differences in any dimension or component summary score. Severity classification was not found to be related to SF-36 scores.

No statistically significant correlation was found between the clinical severity score and SF-36 scores in terms of eight dimensions and two component summary scores (Additional file 2: Table S2).

### Relation between disease control and HRQoL

Among the 104 patients in this study, 35 reported that their edematous attacks were completely controlled (i.e., they experienced no more attacks after disease management), 55 reported that their attacks were partly controlled (i.e., they suffered from fewer attacks after diagnosis and management such as taking long/short-term prophylaxis and avoiding triggers), and 14 reported no improvement of the disease after diagnosis and management (i.e., the disease was not controlled). A correlation between poor disease control and decreased HRQoL scores was found, although the correlation was not significant in terms of RE and MH. The PCS was also significantly correlated with disease control status. Additional file 3: Table S3 shows the correlation between disease control and HRQoL.

In all dimensions except RE, significant differences were found between the three subgroups. Table 2 shows the HRQoL scores in three subgroups (Kruskal-Wallis rank-sum test).

**Table 2:** Median score in the 8 dimensions and 2 component summary scores of SF-36 grouped by disease control status

Dimension	Completely controlled (*n* = 35)	Partly controlled (*n* = 55)	Not controlled (*n* = 14)	*P* value (Kruskal-Wallis test)
PF	100.0 [80.0–100.0]	90.0 [80.0–100.0]	70.0 [58.8–92.5]	*0.004*
RP	100.0 [75.0–100.0]	75.0 [62.5–93.8]	75.0 [48.4–84.4]	*0.010*
BP	80.0 [62.0–100.0]	74.0 [41.0–84.0]	52.0 [38.8–80.5]	*0.044*
GH	52.0 [35.0–72.0]	47.0 [32.0–60.0]	30.0 [10.0–42.8]	*0.003*
VT	68.8 [50.0–75.0]	62.5 [43.8–68.8]	43.8 [25.0–50.0]	*0.002*
SF	87.5 [62.5–100.0]	75.0 [62.5–87.5]	62.5 [37.5–75.0]	*0.016*
RE	75.0 [58.3–100.0]	75.0 [50.0–91.7]	75.0 [50.0–85.4]	0.191
MH	70.0 [55.0–80.0]	70.0 [60.0–75.0]	57.5 [35.0–65.0]	*0.005*
PCS	53.5 [49.0–58.1]	50.0 [45.8–54.4]	43.2 [38.6–52.3]	*0.002*
MCS	45.7 [40.9–51.9]	47.4 [39.8–51.6]	40.9 [34.7–45.5]	*0.028*

The data are summarized as median plus interquartile range [IQR]

Negative correlations between attacks in the 4 weeks before HRQoL measurement and all dimensions of HRQoL were found, although the correlations were not significant in terms of physical functioning (PF), VT and MH. The PCS was also significantly correlated with attacks in the 4 weeks before HRQoL measurement

### Impacts of long-term prophylaxis on HRQoL

In this study, 74 patients were under long-term prophylaxis with danazol. Fifteen patients discontinued danazol prophylaxis due to unbearable side effects or just worrying about side effects. Seventeen patients had never initiated danazol prophylaxis. However, no significant difference was found between patients under long-term prophylaxis and those not under long-term prophylaxis. No correlation was found between HRQoL and danazol dosage when all patients were analyzed on the whole. When the patients were stratified according to sex, the correlations between SF (r = − 0.404, *p* = 0.010), RE (r = − 0.320, *p* = 0.044), MCS (r = − 0.313, *p* = 0.049) and danazol dosage were significant in women.

### Risk factors for low health-related quality of life

We stratified patients into two groups according to the PCS with a cutoff point of 50 and investigated the relationship between the PCS and potential risk factors. HAE patients with current GI edema, laryngeal edema and unsatisfactory disease control had significantly (*p* < 0.05) lower PCSs. A logistic regression model revealed that uncontrolled disease was a risk factor for low PCSs (Table 3).

**Table 3:** Logistic regression analysis of HAE patients with a PCS < 50

	Logistic regression results	
	OR [95% CI]	*P* value
Sex		
Female	2.28 [0.92,5.68]	0.075
Age		
31–44	0.46 [0.15,1.47]	0.190
≥ 45	0.54 [0.130,1.817]	0.374
Onset age		
11–20	0.46 [0.13,1.70]	0.246
≥ 21	0.64 [0.15,2.79]	0.556
Current skin edema		
Positive	0.57 [0.15,2.14]	0.408
Current GI edema		
Positive	1.96 [0.76,5.07]	0.164
Current laryngeal edema		
Positive	2.044 [0.688,6.075]	0.236
Long-term prophylaxis		
Positive	2.73 [0.88,8.41]	0.081
Disease control		
Partly controlled	2.71 [0.82,8.97]	0.102
Not controlled	10.77 [1.78,65.06]	*0.010*

We then stratified the patients into two groups according to the MCS with a cutoff point of 50 and investigated the relationship between the MCS and potential risk factors. HAE patients with current laryngeal edema and unideal disease control had significantly (p < 0.05) lower MCSs. Logistic regression analysis indicated that current laryngeal edema (odds ratio 4.933, 95% confidence interval [CI] 1.154–21.094; p = 0.031) was independently associated with an MCS < 50 in HAE patients (Table 4**.)**.

**Table 4:** Logistic regression analysis of HAE patients with an MCS < 50

	Multivariate logistic regression results	
	OR [95% CI]	p value
Sex		
Female	1.19 [0.43,3.33]	0.740
Age		
31–44	1.82 [0.51,6.50]	0.357
≥ 45	0.64 [0.16,2.53]	0.527
Onset age		
11–20	0.73 [0.16,3.32]	0.683
≥ 21	1.54 [0.28,8.40]	0.615
Current skin edema		
Positive	0.55 [0.14,2.10]	0.379
Current GI edema		
Positive	1.60 [0.54,4.77]	0.396
Current laryngeal edema		
Positive	4.75 [1.09,20.69]	*0.038*
Long-term prophylaxis		
Positive	3.10 [0.95,10.12]	0.061
Disease control		
Partly controlled	2.01 [0.62,6.56]	0.245
Not controlled	1.783 × 10^9^ (0,+ ∞)	0.998

## Discussion

This study is the first to perform a comprehensive analysis of HRQoL in Chinese HAE patients. Since no acute attack medication has been available on the market, the situation in China represents the current status of many countries with limited treatment options. Considering that the general status of Chinese HAE patients has not been evaluated and that condition-specific questionnaires, including AE-QoL and HAE-QoL, do not have validated translated versions, we chose the SF-36 questionnaire to assess the general quality of life in patients. Throughout the study, we found that the HRQoL scores of Chinese HAE patients were significantly lower than those of the general population in all dimensions, which is in accordance with the results in other countries. The population norm was based on a randomized stratified multistage study that included 3,214 people from five geographically representative cities and provided a reliable estimate of the HRQoL in the Chinese population. The HAE cohort in this article has a similar sex ratio to the SF-36 norm cohort (female percentage: 54.81% vs 52.21%). Regarding age composition, the percentage of the 30–39 age group in the HAE cohort was higher (43.27% vs 22.90%) than that in the SF-36 norm cohort, while the percentage of the 60–80 age group was lower (4.81% vs 16.49%). Considering that young people tend to score higher on HRQoL scales [25], the fact that HAE patients reported significantly lower HRQoL scores supported the conclusion that HAE has a negative influence on patient quality of life.

Further subgroup analysis helped us identify patients with decreased quality of life. Women reported lower scores than men in all dimensions except for BP and the PCS. We speculate that estrogen exacerbation may somewhat account for this difference [26]. In addition, women appear to report more adverse reactions to danazol than men [27], which may also contribute to this observation. Patients with mucosal edema have lower RF (i.e., role function) scores than those without. Considering that the main forms of mucosal edema are gastrointestinal edema (usually manifested as severe abdominal pain) and laryngeal edema (manifested as dyspnea or even suffocation), it is reasonable that mucosal edema disturbs daily lives to a large extent. No significant difference was observed between different disease subtypes (type 1 or type 2), probably because of the similar phenotypes in the two forms of C1-INH HAE. The limited number of type 2 patients may have also interfered with the comparison. However, considering that the proportion of type 2 HAE among Chinese patients is lower than that among patients in Europe and America, it was reasonable to include very few type 2 patients in our study. No significant differences were observed between different age groups, which is consistent with the Brazilian study [19]. However, the Swedish study reported a negative correlation between age and quality of life in attack-free periods [28].

In terms of disease severity and HRQoL, no significant correlation was found, which is consistent with previous studies [18, 19]. It may be that because HAE has an extensive influence on the quality of life, even patients who are defined as having mild severity (skin edema less than once per month) would perceive their lives as significantly affected. However, our study found that better disease control was associated with higher SF-36 scores in many dimensions. Physical indicators were more closely related to the disease control state.

We did not observe an association between long-term prophylaxis and HRQoL, which is in accordance with the Swedish study in 2017 [17]. It is thought that patients who do not need long-term prophylaxis usually have much milder disease severity and that their QoL is accordingly less affected by the disease. Among patients who took danazol for long-term prophylaxis, no correlation was found between HRQoL and danazol dosage when all patients were analyzed on the whole. However, considering the side effects of danazol, especially in female patients, it is reasonable to see worse social activity, RE and MH status in female HAE patients who took relatively larger dosages of danazol. Admittedly, the relatively small sample size and the nature of this retrospective study may limit further findings.

Multivariate logistic regression was performed to examine whether sex, age, onset age, disease phenotype and disease control degree were possible predictors of decreased physical or mental component summary scores in SF-36. These factors were selected based on clinical experience and prior literature [17]. Uncontrolled disease was a predictor of PCSs lower than 50 (the normal reference), indicating an association between edematous attacks and impaired physical function. Laryngeal edema was a predictor of MCSs lower than 50 (the normal reference), suggesting that the fear of suffocation may reduce the mental quality of life. Nevertheless, studies using larger samples are needed to find effective predictors for low quality of life scores, thereby providing a reference for therapeutic plans.

There are several limitations in the present study. First, this study included patients who were regularly followed up in a national medical center, which means they may be more seriously ill and may have lower quality of life than other patients. This may have led to an overestimation of HRQoL reduction. Second, only 104 patients were included, although this is an acceptable number considering the rarity of HAE and the difficulty of diagnosis in China. Another limitation is that the study population did not include children, who should be investigated in future research.

## Conclusion

In conclusion, Chinese HAE patients reported significantly lower HRQoL scores than the general population. Unsatisfactory disease control is a risk factor for decreased PCSs. Laryngeal edema is a risk factor for decreased MCSs.

## Methods

### Study population

Patients who presented to the Department of Allergy of the Peking Union Medical College Hospital (PUMCH) with a final diagnosis of HAE type 1 or 2 from 1983 to 2017 were identified through medical records. The inclusion criteria were as follows: 1) a history of recurrent angioedema without urticaria, and/or recurrent attacks of abdominal pain and vomiting, and/or laryngeal edema; 2) decreased C1 inhibitor levels and function confirmed by repeated measurements of type 1 HAE; or 3) normal or slightly increased serum C1 inhibitor levels and lower function confirmed by repeated measurements of type 2 HAE. Although some patients described their family members having similar symptoms, only patients with medical records in our center were included in this study. Among the 400 HAE patients identified, 129 were followed regularly in our center. Most of these patients are probands, which means that they usually present with more severe symptoms than their family members. Two patients younger than 18 years old were excluded from the study. A web-based questionnaire and informed consent were sent to the above 127 patients, and a total of 104 patients completed the web-based questionnaire independently or with the help of family members.

### Questionnaire development

The questionnaire was developed using the wjx online survey platform (www. Wjx.cn). The questionnaire contains mostly close-ended questions with defined response categories. A few questions asked participants to provide descriptive information in an open-ended text box. A pilot questionnaire was sent to 7 volunteering patients to develop the final version. The final version of the questionnaire was sent to the above HAE patients. Participants were informed about the survey, including the purpose, the agency conducting the research and privacy protection. The survey had five sections measuring demographic data, body characteristics, clinical characteristics, socioeconomic issues and HRQoL (using SF-36 for the last).

### Sf-36

SF-36 is a standardized patient-reported survey of generic health measuring eight health domains, including PF, limitations in daily role functioning due to physical problems (RP), BP, general health (GH), vitality (VT), SF, limitations in daily role functioning due to emotional problems (RE), and MH, and it also has an item asking respondents about health changes over the past year. Scores for each domain range from 0 to 100, with higher scores indicating a better health state. Scores on each scale were calculated based on the general survey algorithm. The PCS and the MCS were constructed from eight domains representing PF well-being and emotional well-being. The PCS and MCS were calculated by Optum’s PRO CoRE Software (Pfizer Inc., version 1.3). A standardized Mandarin version of SF-36 was used in this study.

### Clinical severity score

The clinical severity score was expressed using values from 0 to 10 as previously proposed [29]. The score was calculated by considering the age at the onset of disease (0–5 years = 3 points, 6–10 years = 2 points, 11–20 years = 1 point, > 20 years = 0 points), clinical manifestations (skin edema = 1 point, painful abdominal edema = 2 points, laryngeal edema = 2 points, other clinical manifestations = 1 point), and the need for long-term prophylaxis (yes = 1 point).

### Chinese population norm

The Chinese population norm was based on a study by He et al. [25]. A random sample (*n* = 3214) of Chinese adults in five cities of mainland China was collected and analyzed.

### Disease control status

The survey asked patients to report their disease control status in a close-ended question. Disease completely controlled was defined as a patient experiencing no edematous attack at present. Disease partly controlled was defined as a patient experiencing decreased severity after diagnosis and treatment. Disease not controlled was defined as a patient experiencing the same or increased severity after diagnosis with or without treatment.

### Statistical analysis

The data were exported via the wjx online survey platform and were checked through internal error detection. All statistical analyses were performed using SPSS 23.9 (SPSS, Inc., USA) software. Descriptive statistics for demographic information were calculated. The results were expressed as either the mean ± standard deviation [SD] for normally distributed data or the median plus interquartile range [IQR] for nonnormal data. Categorical data were summarized as the percentage of the total group. Differences in quantitative data distributions between patient subgroups were compared using Student’s *t*-test for normally distributed data and by the Wilcoxon rank-sum test or Kruskal-Wallis test for nonnormal data. Differences in frequencies for categorical data were compared using the χ^2^ test. Spearman correlation analysis was applied to explore the correlation between two variables. Full-model logistic regression analysis was used to determine the value of sex, age, onset age, disease phenotype and disease control degree in predicting decreased PCSs and MCSs. A *p* value of < 0.05 was considered statistically significant.

## Additional files

Additional file 1: **Table S1.** Health-related quality of life in Chinese HAE patients. (DOCX 15 kb)

Additional file 2: **Table S2.** Correlation between Clinical Severity Score and HRQoL (Spearman correlation). (DOCX 15 kb)

Additional file 3: **Table S3.** Correlation between disease control and HRQoL (Spearman correlation). (Disease control status: 1 = completely controlled, 2 = partly controlled; 3 = no improvement in disease after diagnosis). (DOCX 15 kb)

Additional file 4: The dataset suppoting the conclusions of this article. (XLSX 21 kb)

## Data Availability

The datasets supporting the conclusions of this article are included within the article and its Additional file 4: Table S4.

## Abbreviations

AE-QoL: angioedema quality of life questionnaire
BP: Bodily pain
C1-INH: Complement 1 inhibitor
CI: Confidence interval
DLQI: Dermatology life quality index
FFP: Fresh frozen plasma
GH: General health
HAE: Hereditary angioedema
HRQoL: Health-related quality of life
IQR: Interquartile range
MCS: Mental component score
MH: Mental health
PCS: Physical component score
PF: Physical functioning
PRO: Patient-reported outcome
RE: Limitations in daily role functioning due to emotional problems
RP: Limitations in daily role functioning due to physical problems
SD: Standard deviation
SF: Social functioning
SF-36: Short-form health survey
VT: Vitality
