# Supplementary material for: Health-related quality of life and its risk factors in Chinese hereditary angioedema patients
Source: Orphanet J Rare Dis. 2019 Aug 8;14:191. doi: 10.1186/s13023-019-1159-5 (PMC6686410; doi:10.1186/s13023-019-1159-5)
Supplement: Supplementary file 1 — Table S1. Health-related quality of life in Chinese HAE patients. (DOCX 15 kb) [file 13023_2019_1159_MOESM1_ESM.docx]

**Supplementary table 1. Health-related quality of life in Chinese HAE patients.**

| Dimension | Chinese HAE patients (n=104) | Chinese norm (n=3214) | p value |
| --- | --- | --- | --- |
| PF | 86.20±16.23 | 94.02±12.44 | p<0.001 |
| RP | 76.38±22.86 | 88.79±28.49 | p<0.001 |
| BP | 66.92±24.54 | 88.18±19.02 | p<0.001 |
| GH | 46.88±21.73 | 69.74±20.95 | p<0.001 |
| VT | 57.75±19.27 | 68.92±18.78 | p<0.001 |
| SF | 72.24±23.30 | 88.03±16.00 | p<0.001 |
| RE | 74.04±22.32 | 89.57±27.95 | p<0.001 |
| MH | 64.76±17.00 | 77.61±15.85 | p<0.001 |
| PCS | 49.81±7.08 |  |  |
| MCS | 44.76±9.18 |  |  |

PF: physical functioning; RP: role-physical; BP: bodily pain; GH: general health; VT: vitality; SF: social functioning; RE: role-emotional; MH: mental health; PCS: physical component score; MCS: mental component score.
