# Supplementary material for: Health-related quality of life and its risk factors in Chinese hereditary angioedema patients
Source: Orphanet J Rare Dis. 2019 Aug 8;14:191. doi: 10.1186/s13023-019-1159-5 (PMC6686410; doi:10.1186/s13023-019-1159-5)
Supplement: Supplementary file 2 — Table S2. Correlation between Clinical Severity Score and HRQoL (Spearman correlation). (DOCX 15 kb) [file 13023_2019_1159_MOESM2_ESM.docx]

**Supplementary table 2. Correlation between Clinical Severity Score and HRQoL (Spearman correlation)**

| Dimension | rs | P value |
| --- | --- | --- |
| PF | -0.029 | 0.770 |
| RP | -0.140 | 0.156 |
| BP | -0.044 | 0.659 |
| GH | -0.015 | 0.882 |
| VT | -0.018 | 0.859 |
| SF | -0.078 | 0.428 |
| RE | -0.050 | 0.611 |
| MH | 0.035 | 0.725 |
| PCS | -0.097 | 0.328 |
| MCS | -0.005 | 0.964 |

rs: Spearman correlation coefficient
