# Supplementary material for: Health-related quality of life and its risk factors in Chinese hereditary angioedema patients
Source: Orphanet J Rare Dis. 2019 Aug 8;14:191. doi: 10.1186/s13023-019-1159-5 (PMC6686410; doi:10.1186/s13023-019-1159-5)
Supplement: Supplementary file 3 — Table S3. Correlation between disease control and HRQoL (Spearman correlation). (Disease control status: 1 = completely controlled, 2 = partly controlled; 3 = no improvement in disease after diagnosis). (DOCX 15 kb) [file 13023_2019_1159_MOESM3_ESM.docx]

**Supplementary table 3. Correlation between disease control and HRQoL (Spearman correlation).** (Disease control status: 1 = completely controlled, 2 = partly controlled; 3 = no improvement in disease after diagnosis)

| Dimension | rs | P value |
| --- | --- | --- |
| PF | -0.310 | *0.001* |
| RP | -0.297 | *0.002* |
| BP | -0.246 | *0.012* |
| GH | -0.291 | *0.003* |
| VT | -0.324 | *0.001* |
| SF | -0.268 | *0.006* |
| RE | -0.179 | 0.070 |
| MH | -0.198 | 0.044 |
| PCS | -0.342 | *＜0.001* |
| MCS | -0.208 | 0.034 |

rs: Spearman correlation coefficient
